# The importance of accurate phenotyping in large-scale analyses of common disorders such as hearing loss

**DOI:** 10.1038/s41431-025-01957-z

**Published:** 2025-09-27

**Authors:** Morag A. Lewis, Bradley A. Schulte, Judy R. Dubno, Karen P. Steel

**Affiliations:** 1 Wolfson Sensory, Pain, and Regeneration Centre, King’s College London, London, UK; 2 The Medical University of South Carolina, Charleston, SC USA

**Keywords:** Diseases of the nervous system, Genetics research

## Abstract

Age-related hearing loss (ARHL) is a common, complex disease with high heritability, but its underlying genetic landscape remains unclear. Studying such a condition requires large cohorts, detailed genotyping, and deep phenotyping. However, while large cohorts with next-generation sequence data are becoming increasingly common, the challenge of administering audiometric tests at scale has meant that in-depth auditory phenotyping is rarely included. Here we present our analyses of three cohorts with different forms of phenotype data, which reveal the differences made by even small changes in phenotyping. Detailed audiometry enables interrogation of genetic data by auditory phenotypes, but if these data are not available, self-reports of hearing difficulty may also serve. However, relying on medical records alone is ineffective for classifying biobank participants for a common condition like ARHL, and is likely to result in many people being wrongly classified in the control group.

## Introduction

Age-related hearing loss is a common, heterogeneous disease with a marked genetic component. Over 150 genes are known to contribute to inherited nonsyndromic human hearing impairment including many with adult-onset [1]. However, the complex contributions of common and rare variants to such conditions are hard to untangle.

The availability of large biobanks has enabled genome-wide association studies for hearing impairment [2–4], and the addition of whole exome and whole genome sequencing to these biobanks has meant that studies can go beyond candidate loci to candidate genes and variants [5, 6]. However, the basic phenotyping of the kind common to large biobanks is a very blunt tool, particularly in the case of conditions such as ARHL which may have a range of underlying pathologies. No large biobanks with general availability offer both deep sequencing and audiometric data, and so investigators must rely on other sources to construct phenotype groups for comparison.

Here we present our comparison of three cohorts, one with detailed audiometric phenotyping, one with carefully-worded questions, and one relying on electronic health records and medical diagnoses. We found that while detailed phenotyping allowed a more in-depth investigation, questions were also effective, as we have previously reported [6, 7]. However, the electronic health records and medical diagnoses appeared insufficient for accurate definition of the control group. This is likely to be a problem for any common condition, particularly those which, like ARHL, typically progress from a mild initial phenotype.

## Methods

### Cohorts

**Fig. 1:**
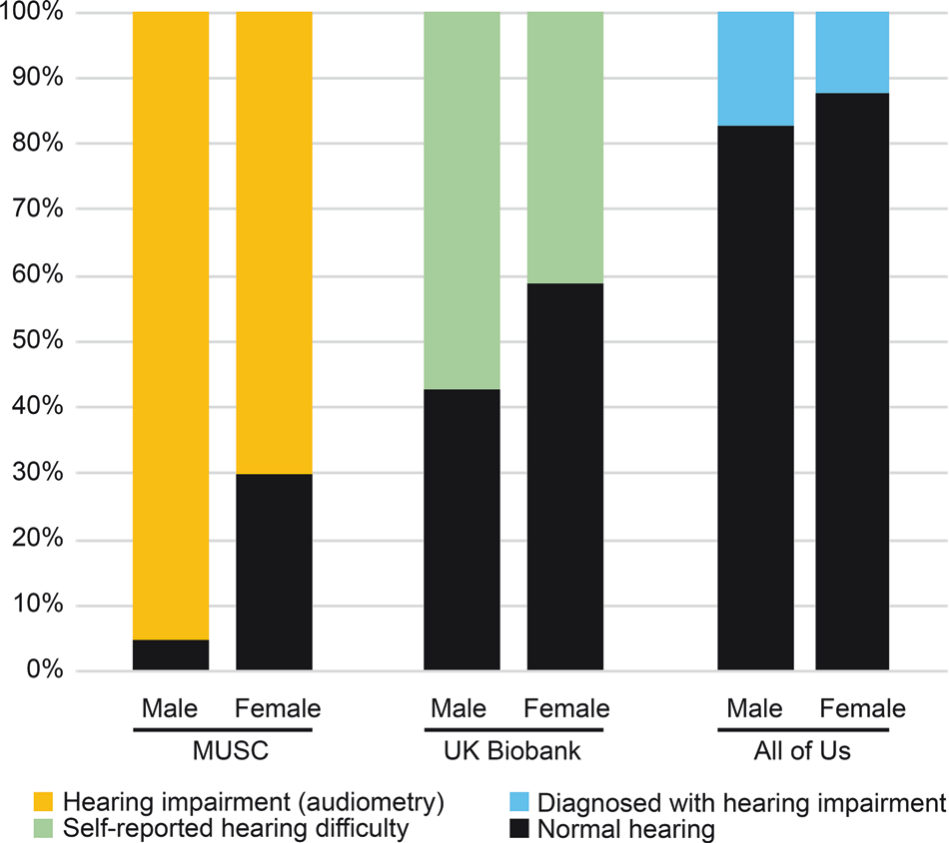
Bar chart showing the proportion of participants with hearing difficulty in the three cohorts. The different methods of phenotyping are shown in yellow (audiometry), green (self-reported hearing difficulty) and blue (diagnostic). Black indicates normal hearing for all three cohorts.

The three cohorts were 1) The Medical University of South Carolina (MUSC) Longitudinal Cohort Study of Age-related Hearing Loss; 2) The UK Biobank; and 3) The National Institutes of Health (NIH) All of Us Research Program. They differ in size, phenotyping and hearing ability (Table 1, Fig. 1). Participants were selected by age (over 55) and absence of other ear diseases (eg Ménière’s Disease). The cohorts and analyses carried out are briefly described below.

1. The MUSC Longitudinal Cohort Study of ARHL is a project focussing on hearing, running since 1987 [8]. Participants are volunteers from South Carolina, and the cohort is skewed towards older adults with hearing loss. They underwent extensive auditory phenotyping and have had their exomes sequenced. Classification by audiometric phenotype is described in [7].
2. The UK Biobank is a very large prospective health study, consisting of 500,000 participants from the UK, aged between 40 and 69 years old in 2006–2010. Around 200,000 participants had exome sequencing when our analysis was carried out. No audiometry was available, so auditory phenotyping was limited to questionnaire responses (Do you have any difficulty with your hearing? Do you find it difficult to follow a conversation if there is background noise? Do you use a hearing aid most of the time?). Classification of participants is described in [6].
3. The NIH All of Us Research Program is a US-based biobank and 245,388 participants had whole genome sequencing at the time of our analysis. No audiometry data were available, so phenotypes were assessed based on electronic health records and survey questions about medical diagnoses (Supplementary Table 1). Sex was based on sex assigned at birth, and participants with entries which were neither “Male” nor “Female” or which did not match recorded gender were excluded from the sex-specific analyses. Predicted genetic ancestry for each participant was provided by All of Us, using previously-described ancestry definitions [9–13].

**Table 1:** Characteristics of the three cohorts described here.

	MUSC	UK Biobank	All of Us
**Size**	532	94,312	93,983
	240 M 292 F	42,472 M 51,840 F	39,073 M 51,522 F
**Ancestry**	European	96% European	57% European
**Phenotyping**	Audiometry	Self-report	Diagnostic coding
**Normal hearing**	99 (19%)	48,731 (52%)	80,343 (85%)
	12 M 87 F	18,235 M 30,496 F	32,320 M 45,103 F
**Hearing impairment**	433 (81%)	45,581 (48%)	13,640 (15%)
	228 M 205 F	24,237 M 21,344 F	6753 M 6419 F

*M* male, *F* female.

### Variant filtering

Variants were annotated using the Ensembl Variant Effect Predictor [14], then filtered by Minor Allele Frequency (MAF) < 10% and high predicted impact on the protein. MAFs vary by population, so for variants assessed in the MUSC cohort, the Non-Finnish European MAF was considered, while for the UK Biobank and All of Us variants, the highest allele frequency for that variant from all populations was considered. Impact was assessed using pathogenicity predictors: CADD [15], SpliceAI [16], and a 5’UTR impact predictor, either Sutr [17] (UK Biobank and MUSC) or 5’UTR Annotator [18] (All of Us).

### Linear regression outlier analysis

A regression analysis was carried out to predict how many variants in each gene would be expected in the hearing-impaired group given the number present in the normal-hearing group. The outlier genes were those with the largest residual differences between predicted and observed variant loads for each gene. The first (Q1) and third (Q3) quartile and the interquartile distance (D = Q3-Q1) were calculated for each regression’s residuals. Genes with residuals <Q1-6D were outlier genes in the normal-hearing group, and those with residuals >Q3 + 6D were outlier genes in the hearing-impaired group [19]. We were interested in outlier genes in the normal-hearing group because variants may protect as well as predispose to hearing impairment.

The outlier gene lists were then compared to a set of 819 genes known to underlie deafness in humans and/or mice (“deafness genes”, Supplementary Table 2 [6, 7]), and to a set of 1213 genes in which variants are commonly reported in sequencing experiments (“variable genes” [6]). Hypergeometric tests were used to assess whether either gene set was significantly enriched in the outlier gene lists. Our hypothesis is that enrichment in deafness genes suggests the outlier list is biologically relevant to hearing, while enrichment in variable genes reflects features unrelated to hearing.

## Results

The outlier analyses of the MUSC cohort and UK Biobank have been previously reported [6, 7]. The deep phenotyping of the MUSC cohort allowed comparisons of different subtypes of ARHL [7] but here we include only the comparison of normal hearing with all hearing impairment, because detailed audiometry was not available for the other two cohorts (Table 2).

**Table 2:** Numbers of outlier genes in the different comparisons of All of Us groups.

		Outliers in hearing difficulty			Outliers in normal hearing		
		All	Male	Female	All	Male	Female
**All of Us**	All participants	554*	547*	302*	1251*	1271*	648*
	African/African American	196*	220	216†*	189*	193*	234*
	American Admixed/Latino	172*	116*	180*	152*	110*	123
	East Asian	668*	-	-	331*	-	-
	European	186	167	159*	144*	131*	133*
	Other	328*	318*	262*	230*	196*	150*
	South Asian	2015	-	-	812†*	-	-
**MUSC**	All participants	96*	-	107*	31	-	35
**UK Biobank**	All participants	**156†**	156	**158†**	222†*	**181†**	**184†**

Not all genetic ancestry groups had sufficient participants to carry out the sex-specific analyses. †Significantly enriched in deafness genes (adj. *p* < 0.05) (highlighted in bold) * Significantly enriched in variable genes (adj. *p* < 0.05). The counts from the UK Biobank cohort and the MUSC cohort are included for comparison; there were not enough male participants in the MUSC cohort to carry out the male-specific analysis (data originally published in [6] and [7]).

The self-reported phenotyping of the UK Biobank meant only one main comparison was carried out, comparing the group with hearing difficulty (who answered “yes” to any of the three questions) to those reporting no hearing difficulty. The size of the cohort meant a robust sex-specific analysis could also be carried out (Table 2) [6].

For the All of Us cohort, the hearing-impaired group was compared to those with no diagnosis of hearing impairment, and the high diversity of participants meant that the comparison could be carried out on some specific genetic ancestry groups as well as sex-separated groups, where the numbers were high enough. Almost all the outlier lists were enriched in variable genes, while only two were enriched in deafness genes (Table 2).

As our prior hypothesis was that ARHL includes a contribution from known deafness genes, the enrichment for these genes in the UK Biobank outlier lists suggested that there was biological validity in the analysis. We did not detect any enrichment for deafness genes among the MUSC cohort outliers, in contrast to our previous findings of significant enrichment in the phenotype-specific analyses [7]. However, the lack of enrichment for deafness genes in most of the All of Us comparisons, coupled with the enrichment of variable genes in nearly every comparison, suggests that these outliers may be chance findings, despite the size of the cohort.

## Discussion

Although the All of Us cohort reports that audiograms have been collected from a subset of participants, these thresholds were not available in the database at the time of our analysis, so our analysis relied upon medical diagnosis in the health records or questions about medical diagnosis. The paucity of deafness gene enrichment in the All of Us cohort may be because many people with ARHL do not obtain medical diagnoses, especially when hearing loss is mild-to-moderate, and thus our “normal-hearing” group in the All of Us cohort is likely to include many people with ARHL. The small size of the hearing-impaired group in the All of Us cohort (15%) bears this out; in the UK Biobank, 48% of the cohort reported difficulty hearing, which concords with the prevalence of the condition in the over-55 age group [20].

Large-scale biobanks have a great deal to offer when it comes to common diseases like hearing loss. Several reports have used the UK Biobank to identify new candidate genes in ARHL [3–6], but all have had to rely on questions about hearing difficulty which do not permit further interrogation of subphenotypes. Furthermore, they are limited to the largely European ancestry of the UK Biobank. For a common condition like ARHL, subjective questions are useful but full audiometry would be better, allowing different pathological forms of hearing loss to be analysed as well as improved definition of the normal-hearing group, which is critically important for any comparison. Better phenotyping and a more diverse cohort should allow more precise identification of the genes and pathways underlying different forms of ARHL.

## Conclusion

In general, in large cohorts with less specific phenotyping, great care is needed to avoid including people with mild or undiagnosed forms of the phenotype under study in the control cohort. The nature of the phenotyping data available must be considered alongside the disease being studied when planning analyses on large cohorts, and consideration given to defining control groups when planning what data to collect in large biobanks. Quantitative audiometric measurements are recommended to maximise the value of large cohorts for investigating hearing impairment.

## Supplementary information

Supplementary Table 1

Supplementary Table 2

## Data Availability

This study used data from the NIH All of Us Research Program’s Controlled Tier Dataset version 7, available to authorized users on the Researcher Workbench.
